# NOD2 dependent neutrophil recruitment is required for early protective immune responses against infectious *Litomosoides sigmodontis* L3 larvae

**DOI:** 10.1038/srep39648

**Published:** 2016-12-22

**Authors:** Jesuthas Ajendra, Sabine Specht, Sebastian Ziewer, Andrea Schiefer, Kenneth Pfarr, Marijo Parčina, Thomas A. Kufer, Achim Hoerauf, Marc P. Hübner

**Affiliations:** 1 Institute of Medical Microbiology, Immunology and Parasitology, University Hospital of Bonn, Bonn, Germany; 2 German Center for Infection Research (DZIF), partner site Bonn-Cologne, Bonn, Germany; 3 Institute of Nutritional Medicine, University Hohenheim, Stuttgart, Germany

## Abstract

Nucleotide-binding oligomerization domain-containing protein 2 (NOD2) recognizes muramyl dipeptide (MDP) of bacterial cell walls, triggering NFκB-induced pro-inflammation. As most human pathogenic filariae contain *Wolbachia* endobacteria that synthesize the MDP-containing cell wall precursor lipid II, NOD2’s role during infection with the rodent filaria *Litomosoides sigmodontis* was investigated. In NFκB reporter-cells, worm-extract containing *Wolbachia* induced NOD2 and NOD1. NOD2-deficient mice infected with *L. sigmodontis* had significantly more worms than wildtype controls early in infection. Increased worm burden was not observed after subcutaneous infection, suggesting that protective NOD2-dependent immune responses occur within the skin. Flow cytometry demonstrated that neutrophil recruitment to the skin was impaired in NOD2^−/−^ mice after intradermal injection of third stage larvae (L3), and blood neutrophil numbers were reduced after *L. sigmodontis* infection. PCR array supported the requirement of NOD2 for recruitment of neutrophils to the skin, as genes associated with neutrophil recruitment and activation were downregulated in NOD2^−/−^ mice after intradermal L3 injection. Neutrophil depletion before *L. sigmodontis* infection increased worm recovery in wildtype mice, confirming that neutrophils are essential against invading L3 larvae. This study indicates that NOD-like receptors are implemented in first-line protective immune responses against filarial nematodes.

Nucleotide-binding oligomerization domain (NOD)-like receptors (NLRs) function as intracellular pattern recognition receptors (PRRs) and thus mediate cytosolic recognition of microbial molecules^1^. The NLR family contains 22 members in humans and 30 in mice^2^, with NOD1 and NOD2 being the best-known and well-studied NLRs (as reviewed in ref. 3). Studies demonstrated that both of these receptors sense bacterial peptidoglycan components, with NOD2 activated by muramyl-dipeptide (MDP), a common peptidoglycan component in Gram-negative and Gram-positive bacteria^4^, whereas NOD1 recognizes di-aminopimelic acid containing peptides (DAP), found in Gram-negative bacteria^5^. Recently, biochemical evidence for the direct binding of MDP to the NOD2 receptor has been reported^6^. Activation of the NOD2 receptor leads to signaling via the proteinkinases RIP2 and TAK1^7^,^8^, culminating in **NFκB** translocation to the nucleus and transcription of pro-inflammatory genes.

NOD2 is expressed in monocytes^9^, dendritic cells^10^, eosinophils^11^, and neutrophils^12^, but also in non-myeloid cell types including intestinal epithelial cells^13^ and keratinocytes^14^. Due to the ability of NOD2 to sense bacterial MDP, its role during bacterial infections and the induction of inflammatory responses has been widely studied. NOD2^−/−^ mice are more susceptible to bacterial infections^15^,^16^ and often respond with an early delayed or impaired neutrophil recruitment phenotype^17^,^18^. A function of NOD2 in murine skin wound healing processes was recently reported, with NOD2 deficiency leading to a substantial defect in wound repair associated with an initial delay in neutrophil recruitment^19^. Furthermore, mutations in the NOD2 gene have been associated with inflammatory bowel diseases like Crohn’s^20^ or inflammatory skin disorder like Blau syndrome^21^.

The role of NOD2 during parasite infections has not been studied as extensively. During infections with the protozoan parasite *Trypanosoma cruzi*, studies indicated that NOD2 is not required for host protection^22^. A proposed role in protection against the intracellular protozoan parasite *Toxoplasma gondii*^23^ was refuted shortly after^24^. Human pathogenic nematodes like *Onchocerca volvulus*, the causative agent of onchocerciasis, or *Wuchereria bancrofti*, the major cause of lymphatic filariasis, are known to harbor endosymbiotic *Wolbachia* bacteria (reviewed in refs 25 and 26). *Wolbachia* are essential for development and survival of their nematode hosts and can induce a pro-inflammatory immune response with strong neutrophil involvement^27^,^28^,^29^,^30^, making them crucial in the pathogenesis of filarial infections^31^,^32^. It has been shown that these Gram-negative bacteria synthesize lipid II, a peptidoglycan precursor that contains MDP^33^, suggesting that *Wolbachia* are a source of NOD2 receptor ligands.

*L. sigmodontis* is a well-established model for human filariasis^34^,^35^, which causes a patent infection in BALB/c mice and develops into adult worms in semi-resistant C57BL/6 mice. Upon natural infection by the intermediate host, the tropical rat mite *Ornithonyssus bacoti*, infectious L3 larvae penetrate the skin and migrate within 5 days via the lymphatics to the thoracic cavity, where they molt into adult worms.

Our experiments demonstrate that crude extracts from *Wolbachia*-containing *L. sigmodontis* adult worms activate both NOD2 and NOD1 *in vitro*. As we had no access to NOD1^−/−^ mice at the time of this study, we focused our *in vivo* experiments on *L. sigmodontis* infections in NOD2^−/−^ mice. The *in vivo* studies revealed that naturally infected C57BL/6 NOD2^−/−^ mice have a higher worm burden during the early and middle phases of infection than wildtype controls. This increased worm burden is due to delayed NOD2-dependent neutrophil recruitment into the skin during the initial phase of the infection. This is the first report demonstrating the impact of an NLR on protective immune responses against a filarial nematode infection, contributing to the still poorly understood protective immune responses within the skin during the initial phase of filarial infection.

## Results

### *Wolbachia* endosymbionts are a possible activator of NOD2 induced signaling

**Figure 1:**
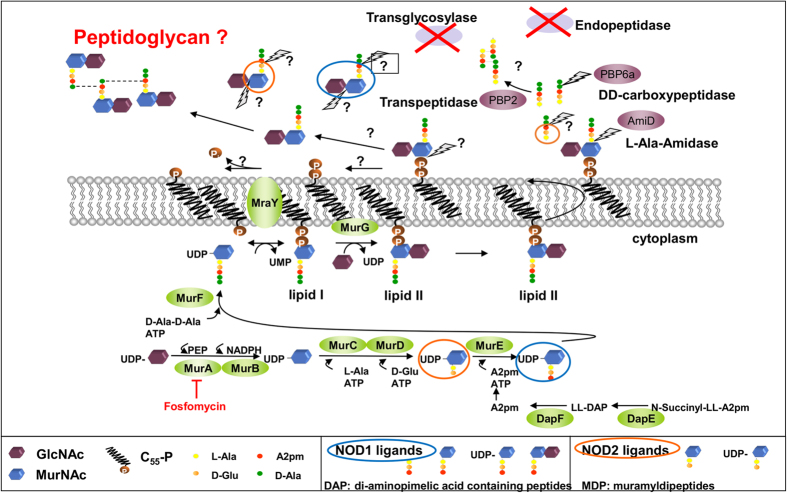
Predicted NOD ligands generated from processing/recycling of *Wolbachia* lipid II. Cytoplasmatic enzymes necessary to produce the peptidoglycan (PG) precursor lipid II are conserved in *Wolbachia* (green ellipses). A2pm is the only amino acid synthesized *de novo*^61^ and it is expected that A2pm is at position three of the peptide chain. MurD and MurE synthesize the NOD ligands UDP-MurNAc-Dipeptide and UDP-MurNAc-Tripeptide, respectively^63^. In the periplasm, *Wolbachia* possess only two penicillin binding proteins (PBPs), a monofunctional transpeptidase and a D-alanyl-D-alanine carboxypeptidase (*Wolbachia* of *Drosophila melanogaster* also encode a monofunctional transpeptidase) that could process the pentapeptide chain^61^,^64^. Transglycosylases and endopeptidases (red X) are absent; therefore “classical” PG with crosslinked glycan chains is not predicted. NOD ligands (blue and orange circles) could arise during recycling or processing (question marks) of lipid II in the periplasmic space. Lightning bolts indicate canonical sites of cleavage in lipid II. MurNAc: N-acetyl-muramic acid, GlcNAc: N-acetyl-glucosamine, C55-P: undecaprenyl phosphate, L-Ala: L-Alanine, D-Glu: D-Glutamic acid, A2pm: diaminopimelic acid, D-Ala: D-Alanine, PBP: Penicillin-binding-protein. Adapted from^33^,^36^.

**Figure 2:**
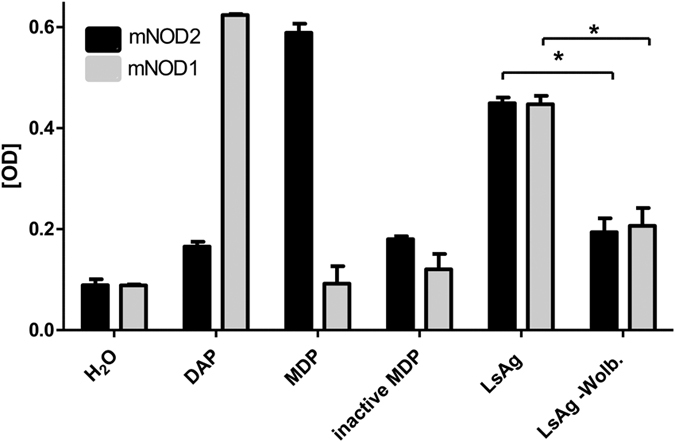
mNOD1 and mNOD2 are activated by complete *L. sigmodontis* crude worm extract. NFκB activation of mNOD1 (grey) and mNOD2 (black) transfected HEK cells stimulated for 10 h with 10 μg/ml *L. sigmodontis* containing (LsAg) or depleted (LsAg-Wolb.) of *Wolbachia* as well as 10 μg/ml of MDP (positive control for NOD2), DAP (positive control for NOD1), inactive MDP (D-D-isomer) and water control. NFκB activation is given in optical density (OD). Representative graph of four independent experiments showing results of quadruplicate samples for LsAg and LsAg-Wolb. and 2–3 samples for the positive and negative controls. Data are shown as mean ± SD and were tested for statistical significance by Mann-Whitney U-test (*p < 0.05).

NOD ligands are derived from lipid II, the precursor of peptidoglycan in bacterial cell walls. It was previously demonstrated that all enzymes needed to synthesize lipid II are encoded in the *Wolbachia* genome (Fig. 1), and that the endobacteria synthesize lipid II^33^,^36^. Thus, the *Wolbachia*, essential endobacteria of filarial nematodes, are potential sources of NOD signaling during filarial infections. To assess whether endosymbiotic *Wolbachia* bacteria of *L. sigmodontis* are indeed activators of NOD1 and NOD2 induced signaling, NFκB-gene-reporter-assays were performed using lysates of *L. sigmodontis* adult worms containing *Wolbachia* or depleted of *Wolbachia*. NOD2-dependent NFκB signaling was induced when HEK293-cells transfected with murine NOD1 or NOD2 genes were stimulated with *Wolbachia-*containing crude extract of *L. sigmodontis* adult worms (Fig. 2), while extract from *Wolbachia*-depleted worms resulted in significantly lower activation of NOD1 and NOD2, respectively. Control stimulations with MDP for NOD2 activation and DAP for NOD1 activation validated the assay.

### Lack of NOD2 results in an increased worm burden

Knowing that the intracellular PRR NOD2 can be activated by *Wolbachia*, the impact of NOD2 signaling on *L. sigmodontis* infection was investigated. To study the role of NOD2 during natural infection with *L. sigmodontis*, the worm burden was quantified at different time points after infection, i.e. after the molt to the L4 stage (15 dpi) as well as a time point when adult worms are present in wildtype (WT) controls (35 dpi).

**Figure 3:**
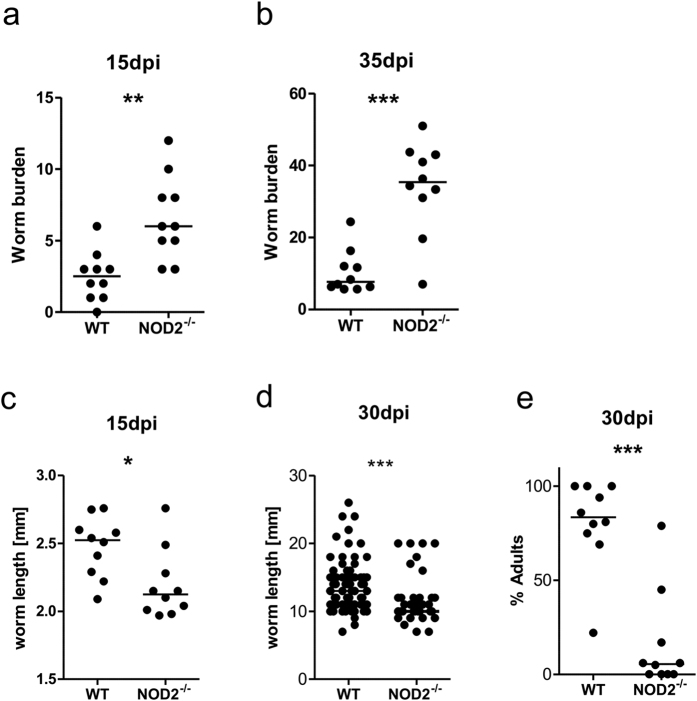
NOD2^−/−^ mice are more susceptible to *L. sigmodontis* infection, but have a retarded worm development. Worm burden in NOD2^−/−^ mice and WT controls 15 dpi (**a**) and 35 dpi (**b**). Length of worms at 15 dpi (**c**) and female worms 30 dpi (**d**). Percentage of *L. sigmodontis* that have undergone molting into the adult stage by 30 dpi (**e**). Data shown as median (**a–e**). The figures are representative of two to three independent experiments with at least 6 mice per group (**a–e**). Statistical significance was calculated with Mann-Whitney U test (*p < 0.05, **p < 0.01, ***p < 0.001).

Compared to WT controls, NOD2^−/−^ mice had an increased worm burden at 15 dpi (Fig. 3a, p = 0.002) as well as at 35 dpi (Fig. 3b, p = 0.001). In addition to the worm burden, length, sex and developmental stage were examined. Worms isolated from NOD2^−/−^ mice were significantly shorter than worms from WT mice (Fig. 3c,d). Furthermore, L4 larvae in NOD2^−/−^ did not molt into the adult stage at the same rate as in WT controls, as by 30 dpi only 15.8% of the L4 larvae had successfully molted into the adult stage in NOD2^−/−^ mice compared to 80.7% in WT mice (Fig. 3e). Differences in sex distribution were not found (data not shown).

**Figure 4:**
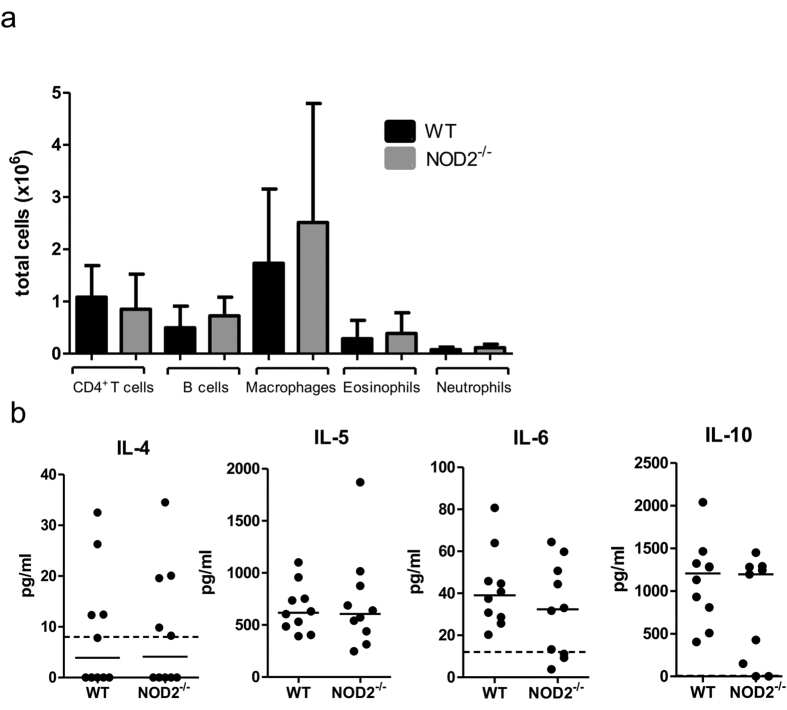
NOD2 deficiency does not impair immune responses at the site of infection. (**a**) Cellular composition of the thoracic cavity 15 days post *L. sigmodontis* infection showing total numbers for the most common cell types of NOD2^−/−^ mice and WT controls. (**b**) Concentrations of IL-4, IL-5, IL-6, and IL-10 within the thoracic cavity lavage 15 days post *L. sigmodontis* infection. Dotted line in (**b**) represents the detection limit of the ELISA. Data are shown as mean with SD (**a**) or median (**b**) and were tested for statistical significance using Mann-Whitney U-test. The graphs are representative of two to three independent experiments with at least 8 mice per group.

The cellular composition and cytokine milieu were also examined at the site of infection, the thoracic cavity, 15 dpi. No differences were found in the absolute numbers of CD4^+^-T cells, B cells, macrophages, eosinophils, and neutrophils in the thoracic cavity of NOD2^−/−^ mice and corresponding WT controls (Fig. 4a). Similarly, cytokine concentrations for IL-4, IL-5, IL-6, and IL-10 in the thoracic cavity lavage did not show significant differences between the two groups (Fig. 4b).

### An increased number of L3 larvae reach the site of infection in NOD2^−/−^ mice

**Figure 5:**
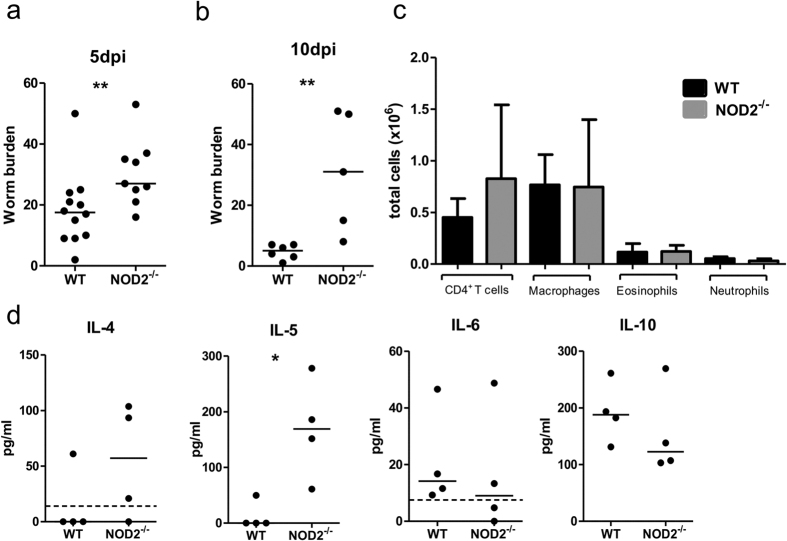
An increased number of L3 larvae reach the thoracic cavity in NOD2^−/−^ mice. (**a**) Worm burden at day 5 post *L. sigmodontis* infection as well as (**b**) 10 dpi. (**c**) Cellular composition of the thoracic cavity lavage 5 dpi in NOD2^−/−^ mice and WT controls. (**d**) Concentrations of IL-4, IL-5, IL-6, and IL-10 within the thoracic cavity lavage 5 dpi. The dotted line in (**d**) represents the detection limit of the ELISA. Data are shown as median (**a,b,d**) or mean with SD (**c**) and were tested for statistical significance using Mann-Whitney U-test. Representative data of two to three independent experiments with 4–12 mice per group.

As the immunological analyses of the thoracic cavity lavage of mice infected for 15 days did not reveal differences that could account for the increased worm burden in the NOD2^−/−^ mice, experiments were performed at 5 dpi, when L3 larvae reach the thoracic cavity, as well as during an early phase of infection (10 dpi). 5 dpi the worm burden in NOD2^−/−^ mice was significantly higher than in WT controls (Fig. 5a, p = 0.008). A significantly higher worm burden was also seen 10 dpi (Fig. 5b, p =  0.0094).

Analysis of the cellular composition and cytokine profile of the thoracic cavity lavage was performed 5 dpi. The overall cell number in the thoracic cavity was lower compared to 15 dpi, with comparable absolute numbers of CD4^+^ T cells, macrophages, eosinophils, and neutrophils between NOD2^−/−^ mice and WT controls (Fig. 5c). Reflecting the reduced total numbers of thoracic cavity cells 5 dpi, cytokine concentrations were lower in both groups compared to 15 dpi. NOD2^−/−^ mice had significantly higher levels of IL-5 5 dpi (Fig. 5d, p =  0.02), while levels of IL-4, IL-6 and IL-10 did not differ compared to WT controls.

### Subcutaneous injection of L3 larvae leads to a similar worm burden in NOD2^−/−^ mice and WT controls

**Figure 6:**
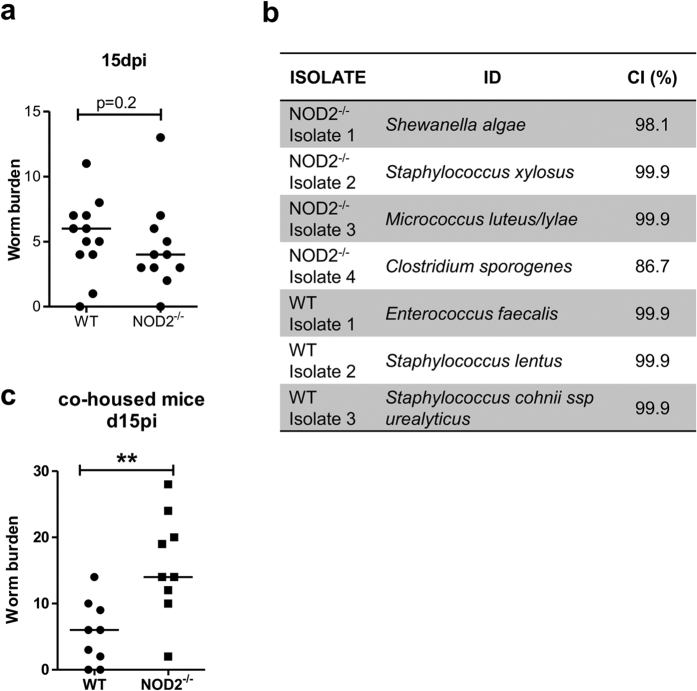
Subcutaneous injection of L3 larvae leads to a similar worm burden in WT and NOD2^−/−^ mice. (**a**) Worm burden at day 15 following s.c. injection of *L. sigmodontis* L3 larvae. Data are shown as median and were tested for statistical significance using Mann-Whitney U-test. The graph represents pooled data from two experiments using 4–6 mice per group. (**b**) Bacterial isolates identified from the skin of WT (n = 5) and NOD2^−/−^ (n = 6) mice. (**c**) Worm burden at 15 days post natural *L. sigmodontis* infection of NOD2^−/−^ and WT mice that were co-housed starting 2 weeks before infection. Data in (**c**) are shown as median and were tested for statistical significance using Mann-Whitney U-test.

Because an increased number of L3 larvae had already reached the site of infection in NOD2^−/−^ mice 5 dpi, we hypothesized that the NOD2 dependent mechanism causing the increased worm burden in NOD2^−/−^ mice must occur within the first few days (<5) of infection. As the skin is one of the major immune barriers in the vertebrate host and has to be penetrated by the L3 larvae during natural infection, the worm burden was assessed 15 days after subcutaneous injection of 40 L3 larvae, thus avoiding the initial trapping of invading L3 larvae within the skin. The worm burden in the thoracic cavity in NOD2^−/−^ mice was not different from that in WT controls (Fig. 6a, p = 0.2; median NOD2^−/−^: 4 worms, median WT: 6 worms), suggesting that the NOD2 dependent protective mechanism occurs within the skin.

Differences in the bacterial colonization of the skin between NOD2^−/−^ and WT mice may account for the observed increased worm burden in NOD2^−/−^ mice. Isolated bacteria from the skin of WT mice consisted of three different types of Gram-positive cocci (Fig. 6b). Flora of NOD2^−/−^ mice included Gram-positive bacteria, but also a Gram-negative rod (*Shewanella algae*), which could be due to contamination of the skin with fecal flora (Fig. 6b). However, differences in the bacterial colonization of the skin seem not to cause the increased worm recovery in NOD2^−/−^ mice, as co-housed NOD2^−/−^ still harbored an increased worm burden 15 dpi compared to the co-housed WT controls (Fig. 6c).

### NOD2 deficiency leads to impaired neutrophil recruitment to the site of infection in the skin

Neutrophils are one of the first immune cells to be recruited to the site of infection and NOD2 deficiency was previously reported to delay neutrophil recruitment^17^,^18^,^19^ following bacterial exposure or in wound healing processes. Therefore, neutrophil counts were analyzed during the initial phase of *L. sigmodontis* infection.

**Figure 7:**
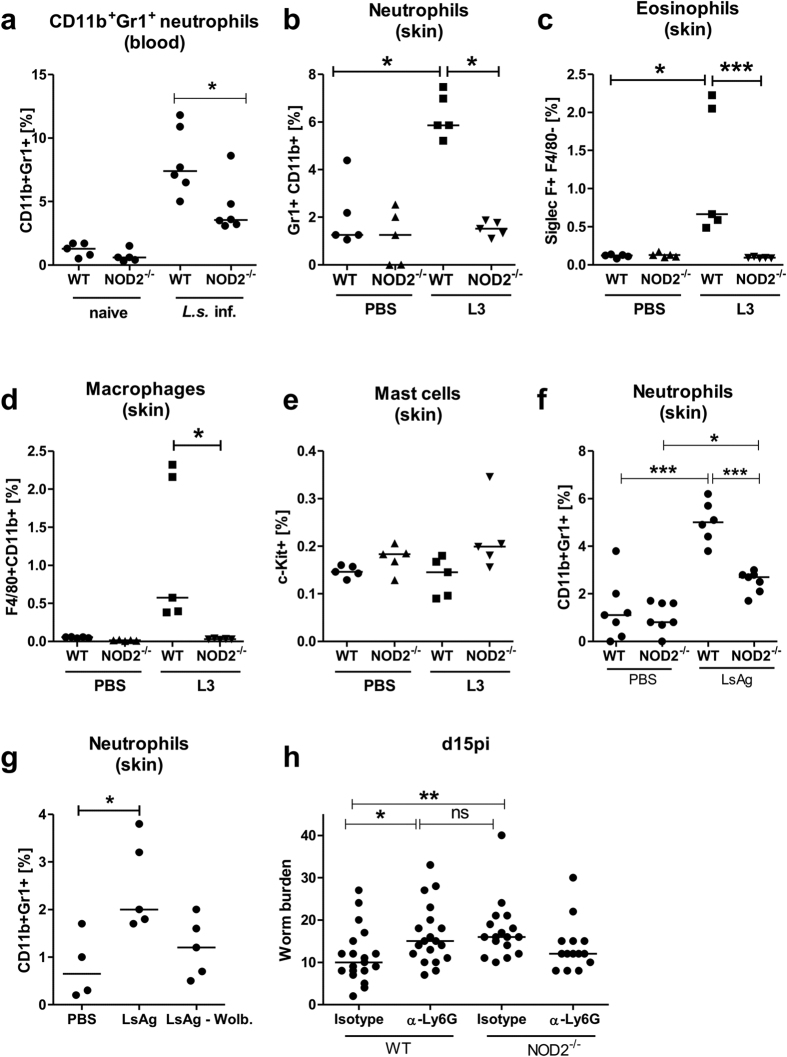
Neutrophils are implemented in the NOD2 dependent protective immune response. (**a**) Frequency of CD11b^+^ Gr1^+^ blood neutrophils in NOD2^−/−^ mice compared to WT mice 24 h post natural *L. sigmodontis* infection as well as in naive mice. Frequency of (**b**) neutrophils, (**c**) eosinophils, (**d**) macrophages, and (**e**) mast cells within the skin 3 h post intradermal L3 or PBS injection in WT and NOD2^−/−^ mice. (**f**) Frequency of neutrophils within the skin 3 h post intradermal LsAg injection in WT and NOD2^−/−^ mice; control groups were injected with PBS. (**g**) Frequency of neutrophils within the skin 3 h post intradermal injection with LsAg, LsAg depleted of *Wolbachia* (LsAg – Wolb.), or PBS in WT mice. (**h**) Worm burden 15 dpi in isotype treated and neutrophil depleted (α-Ly6G) WT and NOD2^−/−^ animals. (**h**) Graph represents pooled data from three independent experiments. Data are displayed as median and were tested for statistical significance by Kruskal-Wallis test followed by Dunn’s post test (*p < 0.05, **p < 0.01, ***p < 0.001).

In comparison to uninfected controls, peripheral blood neutrophil frequencies increased 24 h following natural *L. sigmodontis* infection in both wildtype and NOD2 deficient mice (Fig. 7a). Nevertheless, the increase in neutrophil frequency was significantly lower in infected NOD2^−/−^ mice compared to WT controls (Fig. 7a, p =  0.026). Knowing that neutrophils are involved in the initial immune response and having shown that it is the skin where the NOD2 dependent mechanism occurs, neutrophils and additional innate immune cells: eosinophils, mast cells and macrophages were analyzed from skin via flow cytometry following intradermal L3 injection. Similar to blood neutrophil frequencies following natural infection, intradermal injections of *L. sigmodontis* L3 larvae increased the frequency of CD11b^+^Gr1^+^ neutrophils within the skin compared to PBS treated controls (Fig. 7b). Recruitment of neutrophils in response to L3s was significantly decreased in NOD2^−/−^ mice compared to WT controls (Fig. 7b, p < 0.05). Increased frequencies of eosinophils as well as macrophages were also detected 3 h after L3 injection in WT, but not NOD2^−/−^ mice (Fig. 7c,d), while no differences were observed for mast cells (Fig. 7e). Histological analysis for neutrophil elastase activity further supported the reduced recruitment of neutrophils upon L3 injection in NOD2^−/−^ mice (Suppl. Fig. 1).

Similar to the observed changes upon intradermal L3 challenge, intradermal *L. sigmodontis* extract (LsAg) injections increased the frequency of CD11b^+^Gr1^+^ neutrophils within the skin compared to PBS treated controls and this increase was less prominent in NOD2^−/−^ mice (Fig. 7f, p < 0.001). To investigate whether the neutrophil recruitment to the skin is dependent on *Wolbachia*, additional skin analyses were performed using WT mice and intradermal injections of LsAg as well as extracts prepared from *Wolbachia*-depleted *L. sigmodontis* (LsAg –Wolb.). After injection of LsAg, a significant increase of neutrophils was observed within the skin tissue 3 h post injection compared to the PBS controls. This significant increase in neutrophil frequency was not observed in LsAg depleted from *Wolbachia* injected mice (Fig. 7g).

To further assess the role of neutrophils during *L. sigmodontis* infection, neutrophils were depleted using anti-Ly6G (clone: 1A8) antibody injections 24 h before *L. sigmodontis* infection. Infected WT mice depleted of neutrophils had a significantly increased worm burden in the thoracic cavity 15 dpi compared to isotype-treated WT controls (Fig. 7h). As expected, isotype-treated NOD2^−/−^ mice had a significantly increased worm burden compared to isotype-treated WT controls and neutrophil depletion did not further increase the worm recovery in NOD2^−/−^ mice. In NOD2^−/−^, the observed lower worm burden in the neutrophil depleted animals was not significantly different from the isotype control.

These results indicate that neutrophils are essential in protective immune responses against invading L3 larvae. Neutrophils of NOD2^−/−^ mice exhibit a delayed recruitment to the site of filarial entry, which is associated with the *Wolbachia* endosymbionts of the filarial nematode.

### Neutrophils of NOD2^−/−^ mice are not functionally impaired

**Figure 8:**
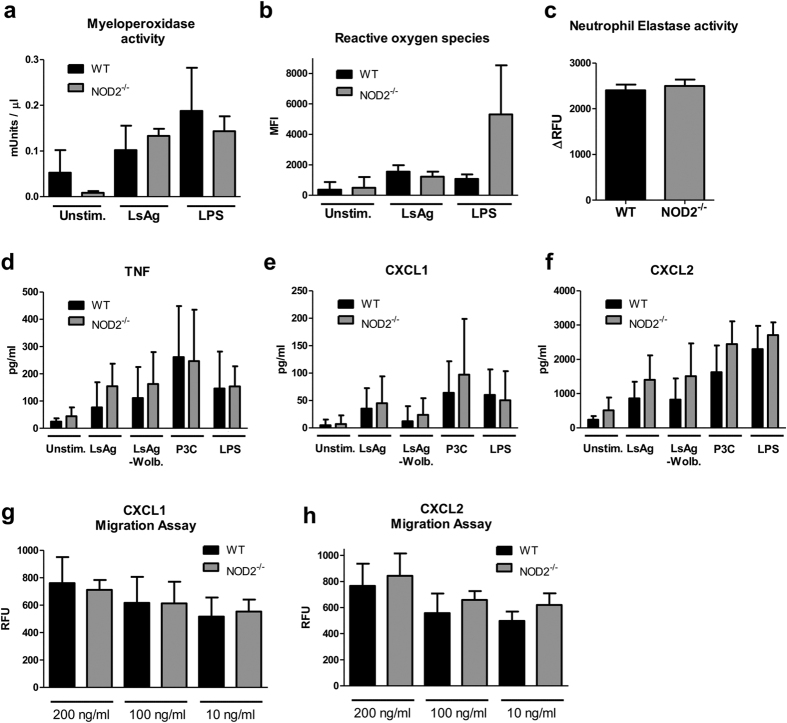
Neutrophils of NOD2^−/−^ mice are not functionally impaired. (**a**) Myeloperoxidase activity and (**b**) reactive oxygen species (ROS) production of casein-induced neutrophils from WT (black) and NOD2^−/−^ mice (grey) after 2 h restimulation with LsAg or LPS as well as unstimulated controls. (**c**) Neutrophil elastase activity of casein-induced neutrophils from WT (black) and NOD2^−/−^ mice (grey). (**d**) TNF, (**e**) CXCL1 and (**f**) CXCL2 production of *in vitro* stimulated neutrophils from NOD2^−/−^ mice and WT controls after stimulation with LsAg, LsAg of *Wolbachia* depleted worms (LsAg - Wolb.), Pam_3_Cys (P3C), and LPS as well as unstimulated controls. (**g,h**) Dose-dependent *in vitro* migration assay using casein-induced neutrophils against the chemoattractants CXCL1 and CXCL2 from WT (black) and NOD2^−/−^ mice (grey). Data are displayed as mean + SD and were tested for statistical significance by Kruskal-Wallis test followed by Dunn’s post test for multiple comparisons (**a,b,d–h**) or Mann-Whitney U-test for comparison of two groups (**c**). Assays were performed with a minimum of 5 mice per group.

To evaluate whether neutrophils of NOD2^−/−^ mice are functionally impaired, casein-induced neutrophils from NOD2^−/−^ and WT mice were compared for their capacity to release diverse pro-inflammatory mediators and their chemotactic behavior. Myeloperoxidase activity and production of reactive oxygen species was comparable for neutrophils from NOD2^−/−^ and WT mice in response to LsAg and LPS (Fig. 8a,b). Similarly, no difference in the activity of neutrophil elastase was observed (Fig. 8c) and the release of TNF, CXCL1 and CXCL2 in response to LsAg, LsAg of *Wolbachia* depleted adult worms, Pam_3_Cys, and LPS were similar (Fig. 8d–f). Furthermore, lack of NOD2 did not alter the dose-dependent *in vitro* migration of neutrophils towards CXCL1 and CXCL2 (Fig. 8g,h). These findings suggest that neutrophils from NOD2^−/−^ mice are not functionally impaired.

### Infectious L3 larvae induce a decreased inflammatory gene expression in NOD2^−/−^ mice

For a more detailed evaluation of immune responses within the skin upon *L. sigmodontis* exposure, a PCR array was performed, measuring the expression of genes known to be involved in immune responses against filariae.

**Figure 9:**
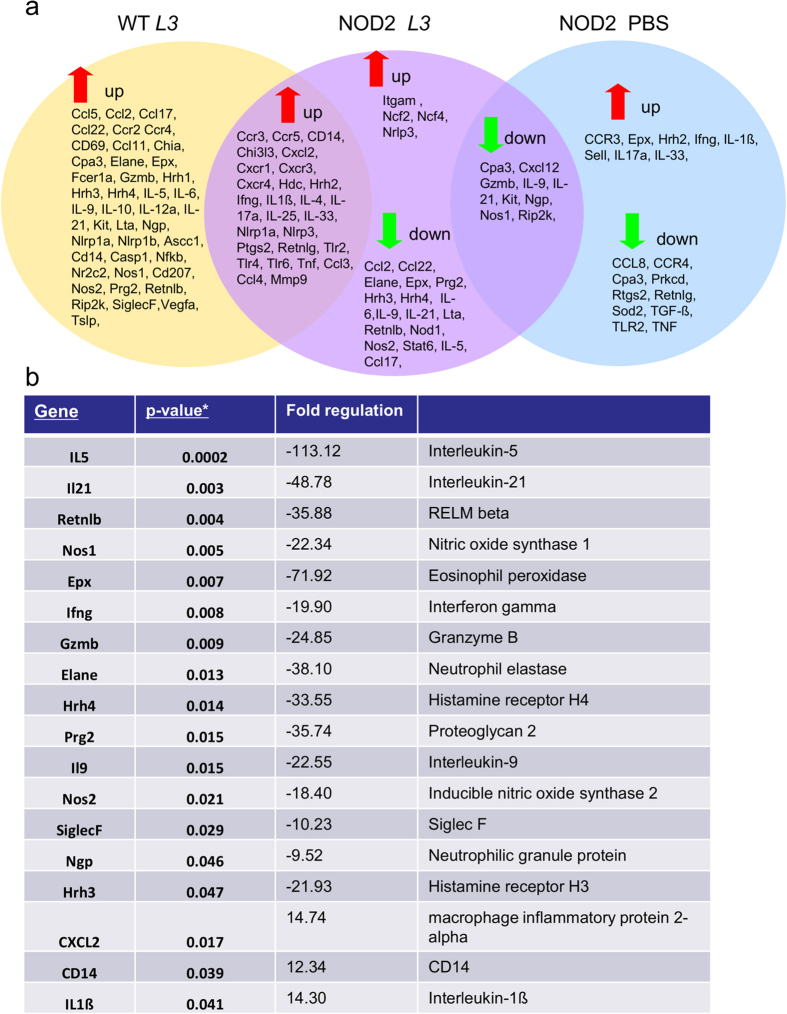
Transcriptional analysis of skin tissue after intradermal L3 injection reveals decreased expression of inflammatory genes in NOD2^−/−^ mice. (**a**) PCR array gene expression profile of skin tissue 3 h post L3 injection, showing investigated genes with decreased or increased expression of all tested groups compared to naive C57BL/6 WT mice. Fold change cut-off for the genes set at >1.5 with three mice per group. (**b**) Genes in skin tissue with significantly decreased expression in NOD2^−/−^ after intradermal L3 injection compared to WT mice after L3 injection. All nominal significant genes are listed and raw p-values as well as fold regulation are given.

NOD2^−/−^ mice and WT controls share a broad range of genes that are upregulated (fold change >1.5) after intradermal L3 injection compared to PBS injected WT controls (Fig. 9a). However, from the PCR array data several genes that are downregulated in L3 injected NOD2^−/−^ mice compared to PBS treated WT controls were identified. None of the measured genes were downregulated in WT mice after L3 injection. When comparing gene expression of L3 injected NOD2^−/−^ mice with L3 injected WT controls, a reduced inflammatory gene expression was detected in the NOD2^−/−^ mice (Fig. 9b).

Genes significantly expressed at lower levels (fold change <−2) in NOD2^−/−^ mice upon L3 injection compared to L3 injected WT controls (Fig. 9b) included *elane*, the gene encoding for neutrophil expressed elastase, *ngp* (neutrophilic granule protein), *nos1* and *nos2* (inducible nitrite oxide synthase I and II), which are also expressed by neutrophils as well as the helminthotoxic protein *prg2* (proteoglycan 2), which is known to have neutrophil activation functions. Differential expression of these neutrophil-associated genes further supports the hypothesis that neutrophils play a major role in the NOD2-dependent mechanism causing the increased worm burden. Interestingly, eosinophil-associated genes encoding eosinophil peroxidase (*epx*) and *siglecf* as well as mast cell-related genes including histamine receptors *hrh3* and *hrh4* were also less strongly expressed in the skin of L3-injected NOD2^−/−^ mice. Furthermore several genes encoding the cytokines *il5, il9, il21*, as well as *ifnγ, gzmb* (granzyme B) and the gene for resistin-like ß (*retnlb*) were significantly upregulated (p-values and fold regulation, see Fig. 9b) in WT controls after L3 injection compared to the NOD2^−/−^ mice (full list of genes with fold regulations: see Supplementary Table 1). qRT-PCR results from skin tissue of L3 injected NOD2^−/−^ mice and WT controls confirmed an increased expression of the eosinophil marker *siglecf* in L3-injected WT controls, while the expression of the mast cell marker *ckit* was not significantly different between the groups (Suppl. Fig. 2a,b). qRT-PCR analysis further demonstrated that L3-injected WT controls had a stronger expression of the pro-inflammatory mediators *inos, il6, gzmb*, as well as the type 2 inducing cytokine *tslp* (Suppl. Fig. 2c–f).

Genes identified in the PCR array that were significantly upregulated in L3-injected NOD2^−/−^ mice compared to L3-injected WT controls included the chemokine *cxcl2*, the LPS detecting co-receptor *cd14*, and *il1ß*, suggesting that inflammatory responses are still occurring in the absence of the NOD2 receptor (Fig. 9b).

Summarizing, the data obtained from the PCR array and qRT-PCR analysis further support our observations on the cellular level, with NOD2^−/−^ mice showing decreased inflammation in the skin compared to WT controls.

## Discussion

Once described as an intracellular PRR exclusively involved in bacterial infections, several studies have suggested that NOD2 has a broader than expected role in the innate immune system^19^,^37^,^38^. Using *L. sigmodontis*, the murine model for human tissue invasive filarial infections, we provide the first evidence that NOD2 is also essential for the early immune response against incoming infectious larvae during infection with filarial nematodes.

Our study demonstrates that NOD2 is crucially involved during the skin stage of infection, as natural infection of NOD2^−/−^ mice with *L. sigmodontis* leads to a significantly increased worm burden during the early phase of infection. This is due to impaired neutrophil recruitment within the skin against incoming infectious L3 larvae, suggesting that the often neglected skin stage of helminth infections is crucial for the establishment of filarial infections.

Previously it was demonstrated that deficiency of NOD2 led to changes in the gut microbiota and increased intestinal inflammation^39^, whereas colonization of NOD2^−/−^ mice with the intestinal helminth *Trichuris muris* inhibited the gut colonization by inflammatory bacterial species^40^. In our study however, differences in the skin microbiota seemed not to mediate the increased worm recovery in NOD2^−/−^ mice, as the increased worm burden in NOD2^−/−^ mice was still present when those animals were co-housed with WT controls. Similar to our results obtained for the skin microbiota, lack of NOD2 (and NOD1) was not associated with changes in the overall composition of the gut microbiota under normal physiological settings^41^.

*Wolbachia* endosymbionts of the nematode seem to be involved in the recruitment of neutrophils to the skin, as injections using crude worm extracts with depleted *Wolbachia* failed to recruit neutrophils. The NOD2 deficiency resulted in an impaired neutrophil recruitment, and was also associated with a decreased expression of inflammatory genes in the skin. This included not only a significantly reduced expression of neutrophil-related genes, but also eosinophil and mast cell associated genes. Taken together with recent publications, our data support that the observed increased worm burden in NOD2^−/−^ is not mediated by eosinophils and mast cells. IL-5 deficient mice which lack eosinophils do not have an increased worm burden 2 and 28 days post *L. sigmodontis* infection^42^, yet IL-5 is required for vaccine induced protection and during the late phase of infection^42^,^43^,^44^. Degranulation of mast cells on the other hand was previously associated with an increased vascular permeability which facilitates *L. sigmodontis* larval migration to the thoracic cavity^45^, suggesting that reduced expression of mast cell associated genes, as observed in NOD2^−/−^ mice, is not the driving factor for the increased worm burden in NOD2^−/−^ mice. However, it should be noted that the mast cell associated genes for the histamine receptors 2 and 4 have also been described to have functions in neutrophil recruitment^46^,^47^,^48^. One neutrophil-associated gene found to be downregulated in NOD2^−/−^ mice was the gene expressing elastase. Histological analysis of neutrophil elastase activity within the skin further confirmed the suppression of neutrophils upon L3 challenge in NOD2^−/−^ mice. This serine proteinase was shown to be part of neutrophil extracellular traps (NETs), which are formed by neutrophils to trap and destroy pathogens^49^. NETs were recently demonstrated to be involved in the defense against incoming larvae of *Strongyloides stercoralis*^50^. In human filarial nematode *Onchocerca volvulus* infections, NET formations were found around the adult worm containing nodules and the mechanism of NET formation was linked to the presence of *Wolbachia* and TLR2/6^51^. The prominent role of neutrophils during the skin phase of *L. sigmodontis* infection in our study is in accordance with the study of Porthouse *et al*., which showed that the very early immune response against incoming *Brugia pahangi* larvae is dominated by neutrophils and IL-6^52^. A recent study by Pionnier *et al*. using neutropenic mice also suggested a protective role of neutrophils during the early stage of *L. sigmodontis* infection^53^. *Wolbachia* endosymbionts are known to induce neutrophil activation and accumulation as shown in the *in vivo* model for ocular onchocerciasis^54^,^55^, in human onchocerciasis^30^ and *in vitro* in which neutrophils are directly activated by human filarial *Wolbachia* lipoproteins^29^. Our data supports the hypothesis that *Wolbachia* derived from incoming L3 larvae provoke neutrophil influx to the skin in a NOD2-dependent manner. As shown by Porthouse *et al*., only about 22% of injected *B. pahangi* L3 larvae are recovered three hours post injection. This high rate of larval death within the first hours of infection is likely to result in a sufficient release of *Wolbachia* to trigger neutrophil recruitment. Keratinocytes, the most abundant cell type of the skin, also express NOD2 and MDP was shown to be the strongest inducer of cytokines in this cell type^7^. Furthermore, phagocytosis of *Wolbachia* by recruited neutrophils can subsequently stimulate production of chemokines and pro-inflammatory cytokines that mediate further neutrophil recruitment and activation^54^. A delayed or impaired neutrophil recruitment in NOD2^−/−^ mice has been described in several studies using bacterial infections^17^,^18^. In those studies, recruited neutrophils were not affected in their function, but the delayed recruitment led to an increased bacterial burden. Similarly, production of pro-inflammatory mediators like MPO, ROS, neutrophil elastase as well as cytokines/chemokines by neutrophils and neutrophil migration towards chemoattractants was not impaired in NOD2^−/−^ mice of our study. Thus, the reduced recruitment of neutrophils to the site of infection within the skin in our study is unlikely due to an impaired chemotaxis of the neutrophils, but rather a reduced inflammatory response at the site of L3 injection.

In theory, resident APCs of the skin can also act as sources of NOD2 activation. However, a study by Cotton *et al*. demonstrated that L3 larvae of *B. malayi* failed to activate *in vitro* generated Langerhans cells and dermal dendritic cells, suggesting that L3 larvae can evade APC detection in human skin at least under *in vitro* conditions^56^. Similar to NOD2, keratinocytes were shown to functionally express NOD1^57^, suggesting that NOD1 may be involved in cutaneous innate immunity. As it was observed that NOD1 also mediates neutrophil recruitment *in vivo* during early stages of immune responses^58^, a lack of the NOD1 gene may also lead to an impaired early immune response against invading L3 larvae in a *Wolbachia*-dependent manner, as our reporter assays also demonstrated *Wolbachia*-dependent NOD1 activation.

In addition to the increased number of infectious larvae reaching the thoracic cavity of NOD2^−/−^ mice, retarded molting and impaired growth of larvae was observed in NOD2^−/−^ mice. This may indicate that the lack of NOD2 may either reduce protective immune responses within the thoracic cavity that were not tested in this study or that immune responses during the migration of the larvae impact their subsequent development. Nevertheless, no microfilaremia developed in NOD2^−/−^ C57BL/6 mice which occurred, for example, in Rag2IL-2Rγ^−/−^ C57BL/6 mice, where the lack of T and B cells leads to an impaired immune response within the thoracic cavity and development of microfilaremia^59^, and the clearance of the filarial infection was comparable in NOD2^−/−^ mice and WT controls.

To our knowledge only two other studies dealt with NOD2 in the context of helminth infections. Using *Trichuris muris* infection, Bowcutt *et al*. demonstrated that NOD2 deficiency leads to an increased worm burden. The authors concluded that the early immune response against *T. muris* is impaired due to the lack of NOD2 dependent recruitment of CD103^+^ dendritic cells, a cell type known to be involved in the clearance of *T. muris*^38^. A study by Wang *et al*. used *T. muris* infection in NOD1/NOD2 double-knockout mice. They demonstrated a significantly delayed parasite expulsion in the intestine of these mice. Their data provides evidence that NOD proteins are triggering goblet cell responses and mucus production in the context of enteric *T. muris* infection^60^. However, in contrast to the filarial nematode *L. sigmodontis, T. muris* does not harbor intracellular bacteria, e.g. *Wolbachia*, that would produce potential NOD2 ligands.

*Wolbachia* are endobacteria classified as Gram-negative, although a “classical” cell wall has not been detected. In the reduced genome of these bacteria, all genes necessary for the enzymatic machinery to synthesize the peptidoglycan precursor lipid II are retained^61^. In Gram-negative bacteria the lipid II molecule is formed by the disaccharide-peptide: GlcNAc-MurNAc-L-Ala-D-Glu-A2pm-D-Ala-D-Ala^62^ and thus contains the precursor for both NOD1 and NOD2 ligands (Fig. 1). NOD1 can recognize several ligands (e.g. GlcNAc-MurNAc-L-Ala-D-Glu-A2pm, MurNAc-L-Ala-D-Glu-A2pm, UDP-MurNAc-L-Ala-D-Glu-A2pm or L-Ala-D-Glu-A2pm) in which the meso-diaminopimelate (A2pm) is necessary for the sensing. By contrast, NOD2 requires the intact sugar molecule MurNAc connected to the dipeptide L-Ala-D-Glu for recognition^63^. The only amino acid that can be synthesized by *Wolbachia de novo* is A2pm^61^,^64^, necessary for the third position of the pentapeptide chain of lipid II and the ligand of NOD1. Using isolated *Wolbachia* membranes, incubated with precursor molecules, we previously showed that lipid II is synthesized *ex vivo*. Additionally, we demonstrated that *Wolbachia* are depleted by fosfomycin, an antibiotic that targets an early step (MurA) in the lipid II production (Fig. 1). Thus the enzymatic pathway for the lipid II synthesis is functional and essential for these endobacteria^33^. The synthesis, recycling and further processing of the lipid II molecule results in the formation of the different NOD ligands and it can be assumed that MDP and A2pm are available in these bacteria (Fig. 1). This is supported by the results herein that indicate that both motifs that trigger NOD2 and NOD1 signaling are present in *Wolbachia* (Fig. 2). Bowcutt *et al*. speculated that NOD2 ligands are expressed on the worm itself. While the *T. muris* studies^38^,^60^ have shown that NOD2 activation does not always rely on the presence of MDP, future studies should investigate which nematode-derived molecules from *Wolbachia*-free species activate NOD2 signaling. Studies with filarial infected humans have not focused on the NOD2 receptor so far. The exception is a study by Babu *et al*., which reported that individuals with filarial lymphedema have a significantly increased expression of NOD2 and NOD1 mRNA after stimulation with *B. malayi* extract compared to asymptomatic individuals^65^. Since our *in vitro* experiments suggested that the *Wolbachia* endosymbionts are also a potential ligand for NOD1 signaling, the role of this receptor should be investigated in the future using NOD1^−/−^ mice.

In conclusion, our study provides first *in vivo* evidence for the involvement of the NOD2 receptor during infection with a filarial nematode. NOD2^−/−^ mice had an increased worm burden due to an impaired early immune response, characterized by significantly lower frequencies of recruited neutrophils in the skin. Hence, NLRs are not only essentially involved in protective immune responses against bacteria and linked to several autoimmune diseases, but may also be key players in the first line defense within the skin against invading infectious larvae.

## Methods

### Ethics Statement

Animal housing conditions and the experiments used in this work were performed according to the European Union animal welfare guidelines. All protocols had been approved by the Landesamt für Natur, Umwelt und Verbraucherschutz, Cologne, Germany (AZ 87-51.04.2011.A025/01; 84-02.04.2014.A327).

### Reporter assays

HEK293 HEK-Blue cells, stably expressing murine NOD1 and NOD2 cells were obtained from Invivogen (San Diego, USA) and maintained according to the manufacturer’s protocol. Briefly, cells were kept in culture with DMEM growth medium containing 4.5 g/l glucose, 10% FCS, 50 U/ml penicillin and 50 μg/ml streptomycin (all from PAA Pasching, Austria). Cells were passaged after reaching 70–80% confluency. To measure NOD1 and NOD2 stimulation, a cell suspension was prepared in HEK-Blue detection medium and added to a plate with *L. sigmodontis* adult worm extract (10 μg/ml), extract of *Wolbachia*-depleted *L. sigmodontis* adult worms (LsAg –Wolb, 10 μg/ml), positive (10 μg/ml MDP and DAP) and negative controls (10 μg/ml inactive MDP, H_2_O). The plate was then incubated at 37 °C in 5% CO_2_ for 10 hours and secreted embryonic alkaline phosphatase (SEAP) reporter protein activity was measured at 650 nm. To lower the background from endogenous alkaline phosphatase, L-homoarginine (Alfa Aesar, Heysham, UK) as a phosphatase-inhibitor was added to cells incubated with the worm extracts.

### Mice and parasites

C57BL/6 J NOD2^−/−^ mice (The Jackson Laboratory, Bar Harbor, ME, USA) and corresponding C57BL/6 J WT controls (Janvier, Saint-Berthevin, France) were housed at the animal facility of the Institute of Medical Microbiology, Immunology and Parasitology, University Hospital of Bonn, Germany. Mice had access to food and water *ad libitum*. Age and sex matched mice were infected at 6–10 weeks of age with *L. sigmodontis* via natural infection by the intermediate host *O. bacoti*. Mice from both groups were exposed simultaneously to the same batch of *O. bacoti* mites containing infectious *L. sigmodontis* L3 larvae^44^.

For subcutaneous injection, L3 larvae were collected after being isolated from dissected mites 11 days post infection and injected subcutaneously.

### Parasite recovery

Mice were euthanized with an overdose of isoflurane (AbbVie, Chicago, USA) 5, 10, 15, 35, and 60 days post infection. The thoracic cavity was flushed with 1 ml of RPMI 1640 (PAA Laboratories, Pasching, Austria) and adult worms or L3/L4 larvae were collected and counted, the sex determined of L4 as well as adult worms and the lengths measured. Measurements of larvae were made using one randomly selected worm per mouse for 15 dpi and 10 female worms per mouse, if present, at 30 dpi. For measurements, the microscope analysis software Diskus 4.6 (Buero Hilgers, Königswinter, Germany) was used.

### Preparation of *L. sigmodontis* antigen

Preparation of *L. sigmodontis* extract (LsAg) was performed as previously described^66^. Briefly, freshly isolated adult *L. sigmodontis* worms were rinsed in sterile PBS before being mechanically homogenization under sterile conditions. Following centrifugation at 300 g for 10 min at 4 °C the supernatant was harvested and the protein concentration was determined by BCA protein assay (Thermo Fisher Scientific, Rockford, USA).

### Flow cytometric analyses of thoracic cavity and skin cells

For analysis by flow cytometry, thoracic cavity cells were obtained by lavage of the thoracic cavity with 5 ml RPMI 1640 medium (PAA Laboratories). The first ml of the lavage was collected and cells were separated from the lavage fluid by centrifugation. After counting, cells were fixed over-night using fixation/permeabilization buffer (eBioscience, San Diego, USA). Cells were then blocked with PBS/1% BSA including 0.1% Fc block (Sigma, St. Louis, USA). Flow cytometric analysis was performed using the following antibody conjugates: CD4-PerCP-Cy5.5, F4/80-APC, SiglecF-PE, Gr1-Pe-Cy7, CD11b-FITC, B220-APC (all eBioscience).

For analysis of cells within the skin, before taking skin samples, hair was removed from a 1 cm × 1 cm area on the upper hind leg region of the mice using depilatory cream. L3 larvae or LsAg were then injected in the center of the shaved region. 3 h post injection, the 1 cm^2^ of skin tissue around the injection site was isolated and treated for 10 min with collagenase (Collagenase P, Roche, Basel, Switzerland) at 37 °C. Digested tissue was forced through a 70 μm strainer. The obtained single-cell suspension was subsequently washed, counted and prepared for flow cytometric analysis as described above. The gating strategy to identify neutrophils from skin tissue is shown in Suppl. Fig. 3.

For analysis of immune cells within the peripheral blood, blood was taken from the facial vein, incubated twice in 1 ml of red blood cell (RBC) lyses buffer (eBioscience) for 5 min, washed with PBS (400 g, 5 min) and prepared for flow cytometric analysis as described above.

Flow cytometry was performed using a BD FACS Canto I system and data were analyzed with the FACSDiva® 5.1 software (BD Biosciences, Heidelberg, Germany). During analysis, gates were set using the FMO (fluorescence minus one) approach.

### Measurement of cytokines

Cytokine concentrations were determined within the first ml of thoracic cavity lavage by ELISA. Cytokines interleukin (IL)-4, IL-5 (both BD Biosciences), IL-6, IL-10, and TNF (all eBioscience) were measured according to kit protocols.

### Neutrophil function assays

For functional assays, neutrophils were isolated from casein injected mice. Casein solution was prepared using casein, PBS, NaOH, 10 mM CaCl_2_ and 5 mM MgCl_2_ and was autoclaved for 1 h at 125 °C. To recruit neutrophils in naïve mice, the casein solution was injected intraperitoneally. Peritoneal cells were isolated 18 h after injection by lavage, pelleted (400 g, 10 min) and counted. To purify neutrophils, peritoneal cells were blocked with PBS/1% BSA including 0.1% rat immunglobulin and then stained for 30 min with Ly6G-PE (clone: 1A8, BioLegend). Cells were then incubated for an additional 15 min with anti-PE MACS antibodies (Miltenyi). Neutrophils were then sorted using positive selection with an autoMACS (Miltenyi Biotech) and purity of Ly6G^+^ neutrophils was checked via flow cytometry, resulting in an average purity of 94%.

To measure the reactive oxygen species (ROS), 2.5 × 10^5^ neutrophils were either unstimulated or stimulated for 2 h with LPS (100 ng/ml) or LsAg (50 μg/ml). After stimulation, cells were incubated per manufacturer’s protocol with CellRox Reagent (Thermo Fisher Scientific) for 30 min at a final concentration of 5 μM. Cells were then washed three times and ROS content was quantified as mean fluorescence intensity using flow cytometry.

Activity of myeloperoxidase (MPO) was analyzed using the MPO colorimetric activity assay kit (Sigma-Aldrich, St. Louis, USA) according to the manufacturer’s protocol, which determines the activity of the enzyme by hydrolysis of substrate to generate taurine chloramine. Samples containing 2 × 10^5^ neutrophils and standard samples were incubated with the MPO substrate and assay buffer for 2 h in a 96-well plate. After stopping the reaction, the plate was measured at 412 nm in an EnVision Multilabel Reader (PerkinElmer, Waltham, MA, USA).

Activity of neutrophil elastase was measured using the neutrophil elastase activity kit (Abcam, Cambridge, United Kingdom). Standard and samples were added to the reaction mix containing neutrophil elastase substrate. After an incubation of 20 min at 37 °C, the activity was measured with a fluorescence microplate reader (EnVision Multilabel Reader, PerkinElmer) at a wavelengths of 380/500 nm (excitation/emission). Neutrophil elastase activity was calculated using a standard curve.

Chemotactic properties of neutrophils were measured using the CytoSelect 96-Well Cell Migration Assay (3 μm, Cellbio Labs). In this assay the cell suspension was placed in the upper chamber and cells were allowed to migrate through the polycarbonate membrane for 2 h towards the chemoattractants CXCL1 and CXCL2 (both Peprotech). Migratory cells cling to the bottom side of the membrane, while non-migratory cells stay in the upper chamber. Migratory cells were then dissociated from the membrane by the addition of a cell detachment buffer to the lower chamber. Migratory cells were then lysed and quantified using a fluorescent dye measured at 480/520 nm (excitation/emission) using an EnVision Multilabel Reader (PerkinElmer).

For cytokine/chemokine measurements, 1 × 10^6^ purified neutrophils per well were cultured in RPMI 1640 medium containing 10% FCS, 1% Penicillin/Streptomycin and 1% L-Glutamine (all from PAA) and stimulated over-night using the following stimuli: *E. coli* LPS (300 ng/ml), Pam_3_Cys (10 ng/ml), LsAg (50 μg/ml), and LsAg prepared from *Wolbachia* depleted filariae (LsAg–Wolb.), both prepared as previously described^67^.

### Neutrophil depletion

Neutrophils were depleted using an anti-Ly6G antibody (clone 1A8, BioXcell, West Lebanon, NH, USA) that was intraperitoneally injected (500 μg/mouse) 24 h prior to infection with *L. sigmodontis*. Control mice were injected with an equal volume of corresponding isotype control (BioXcell). Depletion was confirmed in peripheral blood using an anti-Gr1 (clone RB6-8C5) antibody by flow cytometry.

### Skin microbiome

NOD2^−/−^ and WT mice were shaved on the back and Columbia plates with 5% sheep blood were swabbed over the skin of the mice for half a minute. The plates were incubated for 72 h at 37 °C and 5% CO_2_. All colonies were re-cultured on Columbia 5% sheep blood agar and analyzed by MALDI-TOF (Vitek® MS, BioMeriuex, France) and obtained spectral peeks were analyzed with the SARAMIS^TM^ database (Spectral Archive And Microbial Identification System, BioMérieux).

### Immuno-histochemistry

For immuno-histochemistry, skin tissue was isolated three hours after L3 larvae injection and fixed in 4% formalin. Controls were injected with PBS. Skin tissue was embedded in paraffin and sections were cut at 5 μm. Sections were stained using an anti-mouse neutrophil-elastase kit (Clone NP57, Dako, Hamburg, Germany) using a 1:2000 dilution of the first antibody. Subsequent staining was done according to the manufacturer’s protocol. Afterwards, sections were additionally stained with Hemalaun. Neutrophil elastase activity was assessed by microscopy.

### Gene expression analysis

Skin was isolated 3 h after intradermal injection of *L. sigmodontis* L3 larvae. Negative controls were injected with an equal volume of sterile PBS. Skin samples were homogenized (Precellys24, Peqlab, Erlangen, Germany), and RNA was isolated using Trizol extraction (ThermoFisher) and RNeasy Plus Mini Kit (Qiagen, Hilden, Germany). RNA was quantified using a NanoVue (GE Lifescience, Chalfont St Giles, Great Britain) and quality was assessed using an Experion microfluidic electrophoresis system (Bio-Rad, Hercules, USA). cDNA was synthesized with the RT2 first strand kit (Qiagen). A customized RT2 PCR array (Qiagen, see Supplemental Table 1 for list of genes) was performed on cDNA using a Rotor Gene Q (Qiagen) according to the kit protocol. Data was analyzed and displayed using the online RT2 Profiler PCR Array data analysis 3.5 software on the SABiociences.com website (Qiagen). Gene expression was normalized to three housekeeping genes (*actb, b2m*, and *gapdh*).

To confirm and expand the results from the customized qRT-PCR array analysis, gene expression of selected genes were additionally analyzed using single quantitative real-time-PCRs. cDNA was synthesized as described above and samples were quantified using the PrimePCR™ SYBR® Green Assay (Bio-Rad) according to the manufacturer’s protocol. Gene-specific synthetic DNA templates (Bio-Rad) as a positive PCR control were run simultaneously as standards with the test samples. qRT-PCR were performed using primers for the genes: *ckit* (qMmuCID0021259), *tslp* (qMmuCID0006874), *il6* (qMmuCID0005613), *siglecf* (qMmuCID0026901). The primer pairs used for *gzmb* were “ctgctctgattacccatcgtc” and “gtgaatggacatgaagccagt” and for *inos* “cagctgggctgtacaaaccctt” and “cattggaagtgaagcctttcg”. Gene expression was normalized to the housekeeping gene *actb* (qMmuCED0027505).

### Statistics

Statistical analyses were performed with GraphPad Prism Software Version 5.03 (San Diego, USA). Differences between two unpaired groups were tested for significance with the Mann-Whitney-U-Test. Multiple unpaired groups were tested using Kruskal-Wallis-test with Dunn’s post-hoc test. The statistics from the PCR array data were analyzed using the software provided by SABioscience (Frederick, USA). Further analysis of Δ ct values was done by R (www.r-project.org) and packages from the Bioconductor project (www.bioconductor.org). P-values were calculated using the limma package and corrected for multiple testing using the Benjamini-Hochberg procedure. P-values of <0.05 were considered statistically significant.

## Additional Information

**How to cite this article**: Ajendra, J. *et al*. NOD2 dependent neutrophil recruitment is required for early protective immune responses against infectious *Litomosoides sigmodontis* L3 larvae. *Sci. Rep.* **6**, 39648; doi: 10.1038/srep39648 (2016).

**Publisher's note:** Springer Nature remains neutral with regard to jurisdictional claims in published maps and institutional affiliations.

## Supplementary Material

Supplementary Figures
