# Supplementary material for: NOD2 dependent neutrophil recruitment is required for early protective immune responses against infectious Litomosoides sigmodontis L3 larvae
Source: Sci Rep. 2016 Dec 22;6:39648. doi: 10.1038/srep39648 (PMC5177913; doi:10.1038/srep39648)
Supplement: Supplementary Figures [file srep39648-s1.pdf]

NOD2 dependent neutrophil recruitment is required for early protective immune responses against infectious *Litomosoides sigmodontis* L3 larvae

Jesuthas Ajendra[1,*], Sabine Specht[1,*], Sebastian Ziewer[1], Andrea Schiefer[1], Kenneth Pfarr[1,3], Marijo Parčina[1], Thomas A. Kufer[2], Achim Hoerauf[1, 3, #], Marc P. Hübner[1, #]

[1]Institute of Medical Microbiology, Immunology and Parasitology, University Hospital of Bonn, Bonn, Germany
[2]Institute of Nutritional Medicine, University Hohenheim, Stuttgart, Germany
[3]German Center for Infection Research (DZIF), partner site Bonn-Cologne, Bonn, Germany

[*, #]contributed equally

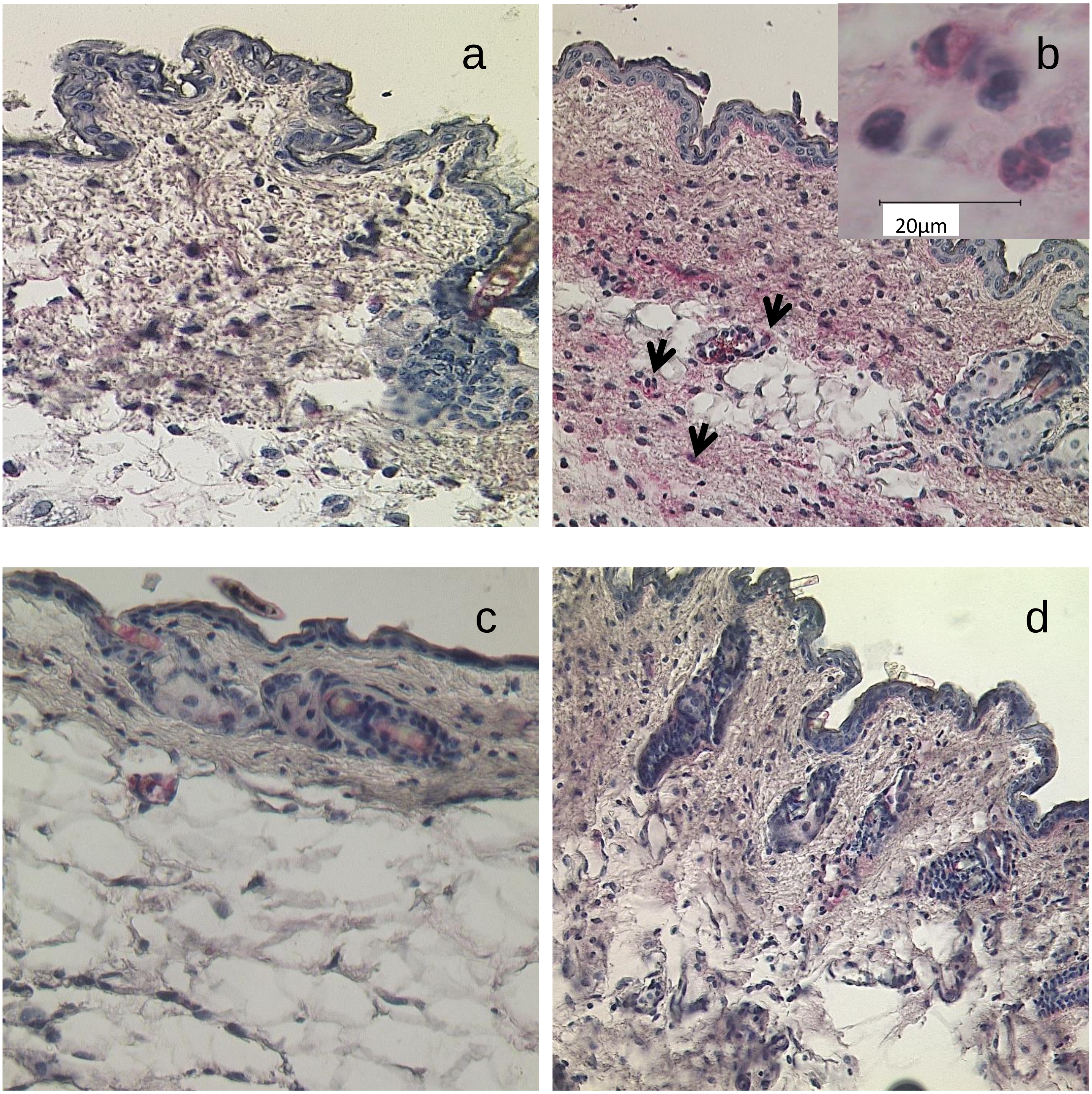

Suppl. Figure 1. NOD2$^{-/-}$ mice have reduced neutrophil elastase activity in the skin tissue after intradermal L3 injection. Representative histological sections of skin tissue 3h post (a) PBS treatment and (b) L3 injection in WT animals as well as (c) PBS treated and (d) L3 injected Nod2$^{-/-}$ animals. Arrows in (b) indicate neutrophils with high elastase activity.

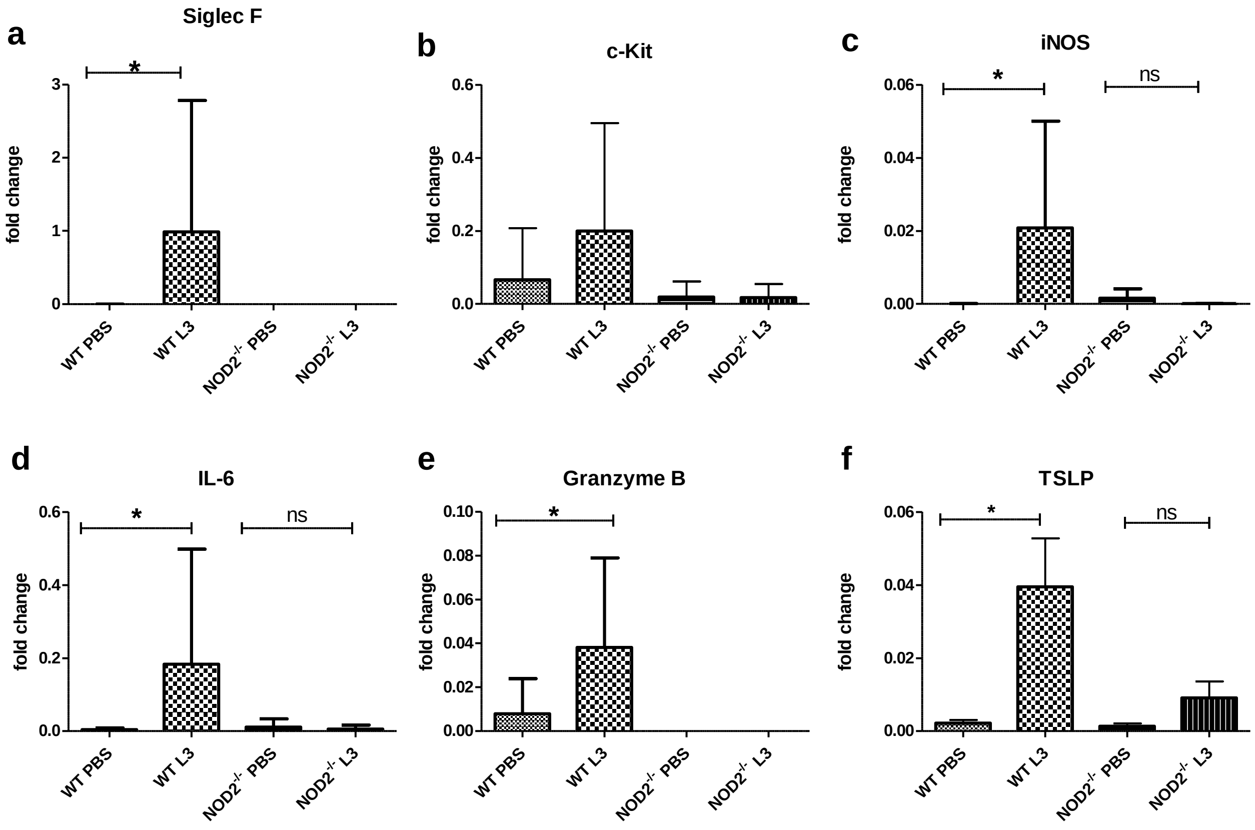

Suppl. Figure 2. NOD2$^{-/-}$ mice have a reduced inflammatory response in the skin tissue after intradermal L3 injection. Gene expression analysis by qRT-PCR of skin tissue 3h post L3 injection in NOD2$^{-/-}$ and WT animals for (a) Siglec F, (b) c-Kit, (c) iNOS, (d) IL-6, (e) Granzyme B, and (f) TSLP. n=6-10 for TSLP, IL-6 and c-Kit; n=3-6 for Siglec F, iNOS and Granzyme B. Shown are means ±SD. Data was tested for statistical significance by Kruskal-Wallis test followed by Dunn‘s post test.

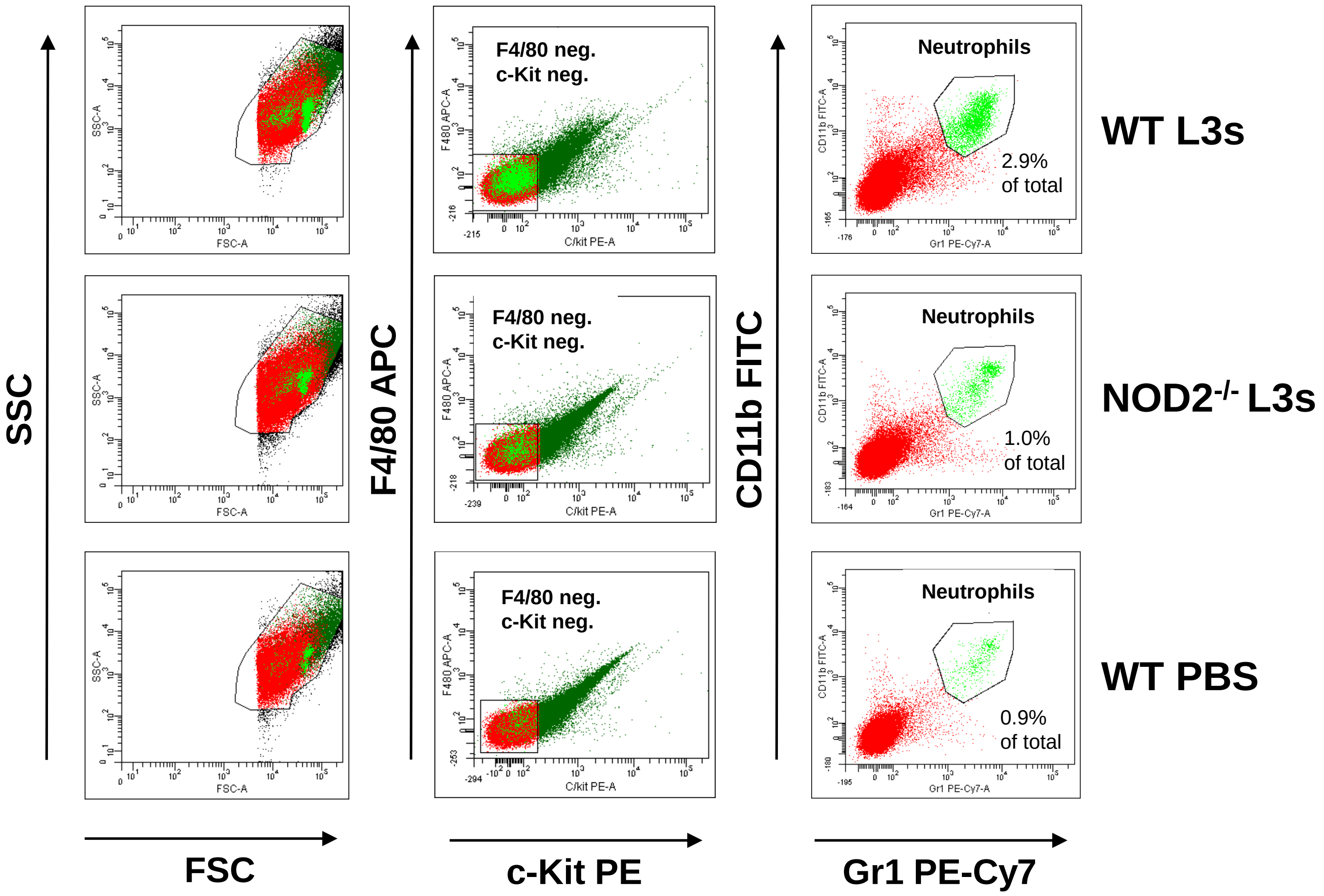

Suppl. Figure 3. Gating strategy to identify Gr1+CD11b+ neutrophils from skin. The neutrophil population was identified based on FSC and SSC characteristics and determined to be c-Kit and F4/80 negative and Gr1 and CD11b positive. Shown are representative figures of samples from skin tissue of L3 or PBS injected WT and NOD2$^{-/-}$ mice.

	Change in gene expression in comparison to PBS treated wildtype controls		
	Wildtype L3	Nod2$^{-/-}$ PBS	Nod2$^{-/-}$ L3
	Fold change	Fold change	Fold change
**Arg1**	0,4682	0,677	0,7766
**Ascc1**	0,9968	0,6631	0,5244
**C3**	0,615	0,6495	0,9991
**Casp1**	0,8228	0,7617	0,5982
**Ccl2**	0,2923	0,4158	0,2336
**Ccl5**	1,4326	0,6116	0,6108
**Ccl8**	0,391	0,2626	1,4359
**Ccl11**	1,426	1,1953	0,4984
**Ccl17**	2,4714	1,1256	0,4319
**Ccl22**	1,1663	0,588	0,2452
**Ccl24**	0,6236	0,6173	0,465
**Ccr2**	1,3122	0,3144	0,5391
**Ccr3**	16,1336	5,4289	8,8275
**Ccr4**	4,3429	0,1789	0,6622
**Ccr5**	3,0356	0,9378	2,5704
**Cd14**	2,0591	1,29	27,3854
**Cd69**	2,5883	0,5538	0,9991
**Cd207**	1,2822	0,9709	0,295
**Chi3l3**	5,7305	0,7868	5,6516
**Chia**	11,7289	1,2403	0,5699
**Cma1**	0,6935	0,5746	1,539
**Cpa3**	0,3839	0,0638	0,1867
**Cxcl2**	4,2732	0,6631	67,8997
**Cxcl12**	0,4481	0,3747	0,6122
**Cxcr1**	9,727	0,4888	7,7382
**Cxcr3**	9,8856	0,7547	9,1177
**Cxcr4**	1,2822	0,8145	2,1765
**Elane**	2,3817	0,8126	0,0674
**Epx**	11,6749	2,2826	0,175
**Fcer1a**	3,8247	0,4255	1,0177
**Il11ra1**	0,5996	0,3339	0,3865
**Gzmb**	1,7355	0,113	0,0753
**Hdc**	2,8128	1,1925	11,8378
**Hrh1**	5,5098	0,859	1,5213
**Hrh2**	16,8967	2,115	3,3219
**Hrh3**	2,8719	0,6361	0,1408
**Hrh4**	8,9507	2,1896	0,2876
**Ifnb1**	38,4612	4,4097	1,1556
**Ifng**	22,5543	3,2132	1,2215
**Il1b**	4,58	1,3293	70,6198
**Il4**	3,447	0,651	6,2854
**Il6**	1,7395	0,6088	0,2856
**Il9**	9,0338	0,4689	0,4319
**Il10**	10,4252	1,1413	0,9518
**Il12a**	13,6611	1,1387	1,6725
**Il17a**	9,3093	2,7524	8,0855
**Il18**	1,2187	1,4784	1,3336
**Il21**	1,6533	0,2177	0,0365
**Il25**	4,6118	0,2775	1,8817
**Il33**	2,5468	2,4748	6,3731
**Il6ra**	0,5342	0,6616	0,6179
**Itgam**	0,8003	0,4026	1,4866
**Kit**	1,1009	0,1814	0,221

**Lta**	2,2325	0,2656	0,3326
**Map3k7**	0,314	0,2233	0,3206
**Ncf2**	1,1371	0,6786	2,29
**Ncf4**	1,2443	1,0526	2,0782
**Nfkb1**	0,8059	0,9844	0,8059
**Nfkb2**	0,6637	0,5131	0,4715
**Ngp**	4,5483	0,3072	0,5148
**Nlrp1a**	11,6479	1,0238	2,5645
**Nlrp3**	9,5931	0,9913	5,8916
**Nod1**	0,7196	0,1708	0,1783
**Nod2**	0,3786	0,4395	0,0731
**Nos1**	3,0497	0,1518	0,1471
**Nos2**	3,8691	0,324	0,2267
**Nr2c2**	0,8096	0,2801	0,5172
**Prg2**	5,035	0,4933	0,1563
**Prkcd**	1,7597	0,1712	1,575
**Ptgs2**	4,7196	0,2995	3,9504
**Retnlb**	7,9926	0,2737	0,2401
**Retnlg**	2,5292	0,1769	46,3769
**Ripk2**	1,456	0,0252	0,0887
**Sell**	7,9742	4,5652	15,0185
**Siglec5**	7,8281	1,4083	0,8247
**Sod2**	0,6164	0,0092	0,0521
**Stat6**	0,8343	0,6245	0,0807
**Tgfb1**	0,5927	0,018	0,3176
**Tlr2**	3,9504	0,6662	2,3598
**Tlr4**	3,5439	0,6259	1,3585
**Tlr6**	3,6689	0,1518	3,3142
**Tnf**	1,9616	0,2228	1,886
**Tslp**	1,5461	0,9913	0,9584
**Vegfa**	2,29	1,6943	2,9053
**IL5**	2,7044	0,099	0,0258
**Mmp9**	1,9525	1,9106	13,6927
**CCL3**	2,1665	1,5881	11,4876
**CCL4**	2,0734	2,2099	21,1903
**Actb**	0,2574	0,3362	0,3865
**B2m**	0,6871	1,0575	0,7163
**Gapdh**	0,943	0,7307	0,8718

Suppl. Table 1. Complete list of genes tested in PCR array analyzing skin tissue three hours post intradermal injection with L3 larvae. As control group served C57BL/6 mice injected with PBS. Three mice per group.
